# Clinical Phenotypic Spectrum and Multisystem Burden in Neurofibromatosis Type 1: A Retrospective Regional Cohort Study

**DOI:** 10.3390/biomedicines14081747

**Published:** 2026-08-03

**Authors:** Aurora Alexandra Jurca, Timea Claudia Ghitea, Claudia Maria Jurca, Cristina Buzlea-Zaha, Dan Bembea, Alexandru Daniel Jurca, Miruna Gug, Emilia Severin, Cosmin Mihai Vesa

**Affiliations:** 1 Faculty of Medicine and Pharmacy, University of Oradea, Universitatii Street 1, 410081 Oradea, Romania; jurca.auroraalexandra@student.uoradea.ro (A.A.J.); frantescu.cristinaadriana@student.uoradea.ro (C.B.-Z.); bembea.dan@student.uoradea.ro (D.B.); 2 Department of Pharmacy, Faculty of Medicine and Pharmacy, University of Oradea, Universitatii Street 1, 410081 Oradea, Romania; 3 Department of Preclinical Disciplines, Faculty of Medicine and Pharmacy, University of Oradea, Universitatii Street 1, 410081 Oradea, Romania; maria.jurca@didactic.uoradea.ro (C.M.J.); alexandru.jurca@didactic.uoradea.ro (A.D.J.); vesa.cosmin.mihai@didactic.uoradea.ro (C.M.V.); 4 Doctoral School “Victor Babes”, University of Medicine and Pharmacy Timisoara, Eftimie Murgu Square 2, 300041 Timisoara, Romania; 5 Department of Genetics, University of Medicine and Pharmacy “Carol Davila” Bucharest, Dionisie Lupu Street, Number 37, District 2, 020021 Bucharest, Romania; emilia.severin@umfcd.ro

**Keywords:** neurofibromatosis type 1, NF1, neurocutaneous syndrome, café-au-lait macules, plexiform neurofibroma, multisystem burden, extracutaneous involvement, disease evolution, clinical phenotype, regional cohort

## Abstract

**Background**: Neurofibromatosis type 1 (NF1) is a multisystem genetic disorder characterized by marked phenotypic heterogeneity. This study aimed to describe the clinical spectrum and multisystem burden of NF1 in a regional retrospective cohort. **Methods**: We retrospectively analyzed 77 unique patients with NF1 after removal of duplicate records. Demographic characteristics, cutaneous manifestations, extracutaneous involvement, therapeutic interventions, and disease evolution were evaluated. An exploratory multisystem burden score was calculated using six extracutaneous clinical domains. **Results**: Café-au-lait macules were present in all patients, axillary/inguinal freckling in 93.5%, cutaneous neurofibromas in 63.6%, and plexiform tumors in 28.6%. Psychiatric/behavioral (67.5%), skeletal (57.1%), ophthalmological (51.9%), and neurological (45.5%) involvement were common. More than half of the cohort had involvement of at least three extracutaneous domains. Plexiform tumors were more common in patients with progressive or unfavorable disease compared to those with stationary disease. Progressive or unfavorable evolution was associated with a higher frequency of plexiform tumors. Therapeutic interventions were reported descriptively. **Conclusions**: NF1 showed substantial multisystem involvement beyond its characteristic cutaneous manifestations. Plexiform tumors were associated with a more complex clinical course. The proposed exploratory multisystem burden score may facilitate descriptive assessment of disease breadth but requires prospective validation.

## 1. Introduction

Neurofibromatosis type 1 (NF1) is one of the most common autosomal dominant neurocutaneous syndromes and represents a complex multisystem genetic disorder with highly variable clinical expression. Its estimated prevalence is commonly reported to be around 1 in 3000–4000 individuals, although epidemiological estimates vary according to population and methodology. NF1 is caused by pathogenic variants in the *NF1* gene, which encodes neurofibromin, a tumor suppressor protein involved in negative regulation of RAS signaling. Loss of neurofibromin function contributes to abnormal cell growth, tumor predisposition, and a broad spectrum of developmental and non-tumor manifestations [1,2].

Clinically, NF1 is characterized by pigmentary changes, peripheral nerve sheath tumors, ophthalmological manifestations, skeletal abnormalities, neurodevelopmental difficulties, behavioral disorders, and an increased risk of benign and malignant tumors. Classical hallmark manifestations include multiple café-au-lait macules (CALMs), axillary or inguinal freckling, cutaneous neurofibromas, plexiform neurofibromas, Lisch nodules, optic pathway gliomas, and distinctive osseous lesions. However, the clinical spectrum is highly heterogeneous, even among affected individuals from the same family, complicating prognosis and follow-up planning [3,4,5].

The diagnosis of NF1 has traditionally relied on clinical criteria, particularly pigmentary and tumor-related manifestations. In 2021, international consensus recommendations revised the diagnostic criteria for NF1 and Legius syndrome, incorporating new clinical features and molecular testing to improve diagnostic precision, especially in young children and in patients with overlapping pigmentary phenotypes. This update is clinically important because early pigmentary manifestations may precede tumor-related or extracutaneous complications, while genetic testing can support diagnosis in uncertain cases and assist genetic counseling [6,7].

Although CALMs and freckling are frequently the earliest and most visible signs, NF1 should not be regarded as a purely dermatological condition. It is a lifelong disorder with potential involvement of multiple organs and systems. Skeletal abnormalities, including scoliosis and other bone deformities, may appear during childhood and adolescence and can require orthopedic follow-up. Ophthalmological manifestations, particularly Lisch nodules and optic pathway gliomas, remain important diagnostic and surveillance targets. Neurological complications, neurodevelopmental impairment, language difficulties, learning disabilities, attention-related symptoms, anxiety, and behavioral disturbances may substantially affect quality of life, educational performance, and social functioning [8,9].

Plexiform neurofibromas represent one of the most clinically relevant NF1-associated tumor manifestations. These lesions may be congenital or develop early in life and can cause pain, disfigurement, functional limitation, neurological deficits, and compression of adjacent structures. They are also important because a subset may undergo malignant transformation into malignant peripheral nerve sheath tumors. Consequently, the presence of plexiform neurofibromas is a key marker of clinical complexity and long-term surveillance needs. Current tumor surveillance recommendations emphasize structured clinical follow-up across the lifespan, with special attention to tumor-related complications in both pediatric and adult NF1 patients [5,10,11].

The therapeutic landscape of NF1 has also evolved. Historically, management was mainly based on surveillance, symptomatic treatment, surgical intervention, rehabilitation, and management of complications. More recently, targeted therapy with MEK inhibitors, particularly selumetinib, has introduced a new treatment option for selected patients with symptomatic, inoperable plexiform neurofibromas. The pivotal phase 2 trial of selumetinib in children with NF1 and inoperable plexiform neurofibromas reported durable tumor shrinkage and clinical benefit, supporting the transition toward more individualized and molecularly informed management strategies [12,13].

Despite increasing knowledge regarding NF1, real-world regional data remain important because the clinical burden of the disease is influenced by referral patterns, access to genetic testing, multidisciplinary surveillance, imaging availability, and age at diagnosis. In many clinical settings, patients are followed across different specialties, and the cumulative multisystem burden may be underestimated if clinical information is fragmented. Therefore, integrated cohort analyses are useful for characterizing the phenotypic spectrum of NF1 and identifying clinical patterns associated with disease severity, therapeutic burden, and progressive evolution [14,15].

The present study primarily aimed to introduce and apply an exploratory multisystem burden score that summarizes cumulative extracutaneous involvement in NF1 using six clinically relevant domains. Secondary objectives were to describe the regional clinical phenotype and to examine whether a broader multisystem burden and plexiform tumor involvement were associated with progressive or unfavorable disease evolution. The score was designed as a pragmatic descriptive instrument for retrospective clinical datasets and was not intended to replace established diagnostic criteria or organ-specific severity measures.

## 2. Materials and Methods

### 2.1. Study Design and Setting

This retrospective observational study included patients with NF1 evaluated at the Bihor Regional Center for Medical Genetics, Bihor County Emergency Clinical Hospital, between 1990 and 2025. Clinical records were reviewed to characterize phenotype, extracutaneous involvement, treatment, and disease evolution.

The study followed a descriptive and analytical design. First, the demographic, familial, cutaneous, tumor-related, ophthalmological, neurological, skeletal, neurodevelopmental, psychiatric/behavioral, cardiac, imaging, therapeutic, and evolutionary characteristics of the cohort were summarized. Second, patients were stratified according to disease evolution in order to explore clinical and therapeutic markers associated with progressive or unfavorable disease.

### 2.2. Study Population

The initial database contained 101 entries. After removal of 24 exact duplicates, 77 unique patients with a documented NF1 diagnosis and sufficient clinical information were retained. Pediatric and adult patients were included.

Patients were eligible for inclusion if they had a recorded diagnosis of NF1 and available clinical information regarding demographic characteristics and at least one NF1-related clinical domain. Patients with duplicate records were retained only once. Entries with insufficient clinical information for analysis or inconsistent duplicated data were excluded from the final analytical dataset.

The final cohort included both pediatric and adult patients, allowing for evaluation of NF1 manifestations across a broad age spectrum.

### 2.3. Data Collection and Variables

Clinical records were reviewed to extract demographic characteristics, family history, cutaneous manifestations, tumor-related findings, extracutaneous involvement, imaging findings, therapeutic interventions, and disease evolution.

Cutaneous variables included café-au-lait macules, axillary or inguinal freckling, cutaneous neurofibromas, and plexiform tumors, together with their recorded number, localization, and age at onset. Plexiform tumors were considered present when directly documented or when at least one corresponding localization, number, or age-of-onset variable indicated their presence.

Extracutaneous manifestations were recorded according to the predefined clinical domains described in Section 2.5. Imaging abnormalities documented on CT, MRI, or abdominal ultrasound were also collected when available.

Therapeutic variables included surgical treatment, number of surgical interventions, symptomatic medical treatment, physiokinetotherapy/rehabilitation, and selumetinib therapy.

Disease evolution and family history were extracted from the clinical database. Family relationships between affected individuals could not be reconstructed because pedigree linkage was not consistently available.

### 2.4. Definition of Clinical Domains

Extracutaneous manifestations were subsequently grouped into the predefined domains described in Section 2.5. A domain was coded as affected when at least one corresponding manifestation was documented; cutaneous diagnostic features were analyzed separately.

Each domain was coded as positive if at least one manifestation belonging to that domain was present. For example, ophthalmological involvement was considered present if the patient had Lisch nodules, optic pathway glioma, glaucoma, or another recorded ophthalmological abnormality. Similarly, neurological involvement was considered present if any neurological manifestation was documented.

Cutaneous manifestations, including café-au-lait macules, freckling, and cutaneous neurofibromas, were analyzed separately because they represent hallmark diagnostic features of NF1 rather than extracutaneous disease burden.

### 2.5. Multisystem Burden Score

An exploratory multisystem burden score was calculated for each patient to provide a single, transparent measure of cumulative extracutaneous involvement. Six domains were selected a priori because they were consistently represented in the clinical database and reflect major non-cutaneous components of NF1 care: skeletal, ophthalmological, neurological, neurodevelopmental, psychiatric/behavioral, and cardiac involvement.

Each domain contributed one point when at least one manifestation was documented and zero points when no manifestation was recorded. The unweighted total therefore ranged from 0 to 6. An unweighted approach was chosen to preserve interpretability and to avoid assigning unsupported severity weights in a retrospective dataset. For descriptive stratification, involvement of three or more domains was classified as high multisystem burden.

The score was used to summarize the breadth, not the intensity, of extracutaneous disease and to compare overall involvement between evolution groups. It should be regarded as hypothesis-generating because it has not undergone external validation, reliability testing, or prospective assessment of predictive performance.

### 2.6. Disease Evolution Groups

Disease evolution categories were extracted directly from the longitudinal clinical records maintained at the Regional Center for Medical Genetics. The original clinical classification reflected the overall medical judgment documented during follow-up. In general, stationary evolution indicated stable disease without clinically relevant progression, progressive evolution indicated the appearance or enlargement of NF1-related manifestations requiring closer follow-up or therapeutic intervention, unfavorable evolution referred to clinically significant deterioration associated with increased morbidity or complications, whereas favorable evolution indicated clinical stability or improvement during follow-up. Because this was a retrospective study, these categories were based on routine clinical documentation rather than predefined research criteria.

This grouping was used to evaluate whether clinical severity markers, plexiform tumors, therapeutic interventions, and multisystem burden differed according to disease course.

### 2.7. Imaging and Therapeutic Assessment

CT, MRI, and abdominal ultrasound abnormalities were recorded when available. Therapeutic variables included surgery, symptomatic treatment, rehabilitation, and selumetinib; these data were interpreted descriptively because lesion-specific treatment indications were not consistently documented.

Therapeutic burden was assessed using several variables: documented surgical treatment, number of surgical interventions, medical treatment, physiokinetotherapy/rehabilitation, selumetinib therapy, and any therapeutic intervention. Any therapeutic intervention was defined as the presence of at least one of the following: surgical treatment, medical treatment, physiokinetotherapy/rehabilitation, or selumetinib therapy.

### 2.8. Statistical Analysis

Statistical analysis was performed using the cleaned patient-level dataset after removal of duplicate entries. Categorical variables are reported as *n* (%), and continuous variables as mean ± SD or median (IQR/range), as appropriate. Fisher’s exact test was used for categorical comparisons and the Mann–Whitney U test for continuous variables. Analyses were exploratory, with *p* < 0.05 considered statistically significant; SPSS version 30 was used.

Comparisons between patients with stationary disease evolution and those with progressive or unfavorable disease evolution focused on plexiform tumor frequency, age variables, and the exploratory multisystem burden score. Fisher’s exact test was used for categorical variables and the Mann–Whitney U test for continuous or ordinal variables because of the relatively small sample and non-normal distributions. Therapeutic interventions were reported descriptively and were not treated as independent markers of disease evolution because treatment need is inherently influenced by clinical progression and lesion-related morbidity.

A *p*-value < 0.05 was considered statistically significant. Given the retrospective and exploratory nature of the study, results were interpreted descriptively, with emphasis on clinical relevance and effect patterns rather than causal inference.

All analyses were performed using SPSS software (Version 30, Armonk, NY, USA). Figures were generated from the final analytical dataset of 77 unique patients.

### 2.9. Ethical Considerations

The study was conducted in accordance with the principles of the Declaration of Helsinki. As this was a retrospective analysis of existing clinical data, no additional procedures were performed for research purposes. Patient data were anonymized prior to analysis, and direct personal identifiers were removed from the working dataset.

The study was approved by the Ethics Committee of University of Oradea (approval number 33, date: 30 July 2025).

**Figure 1:**
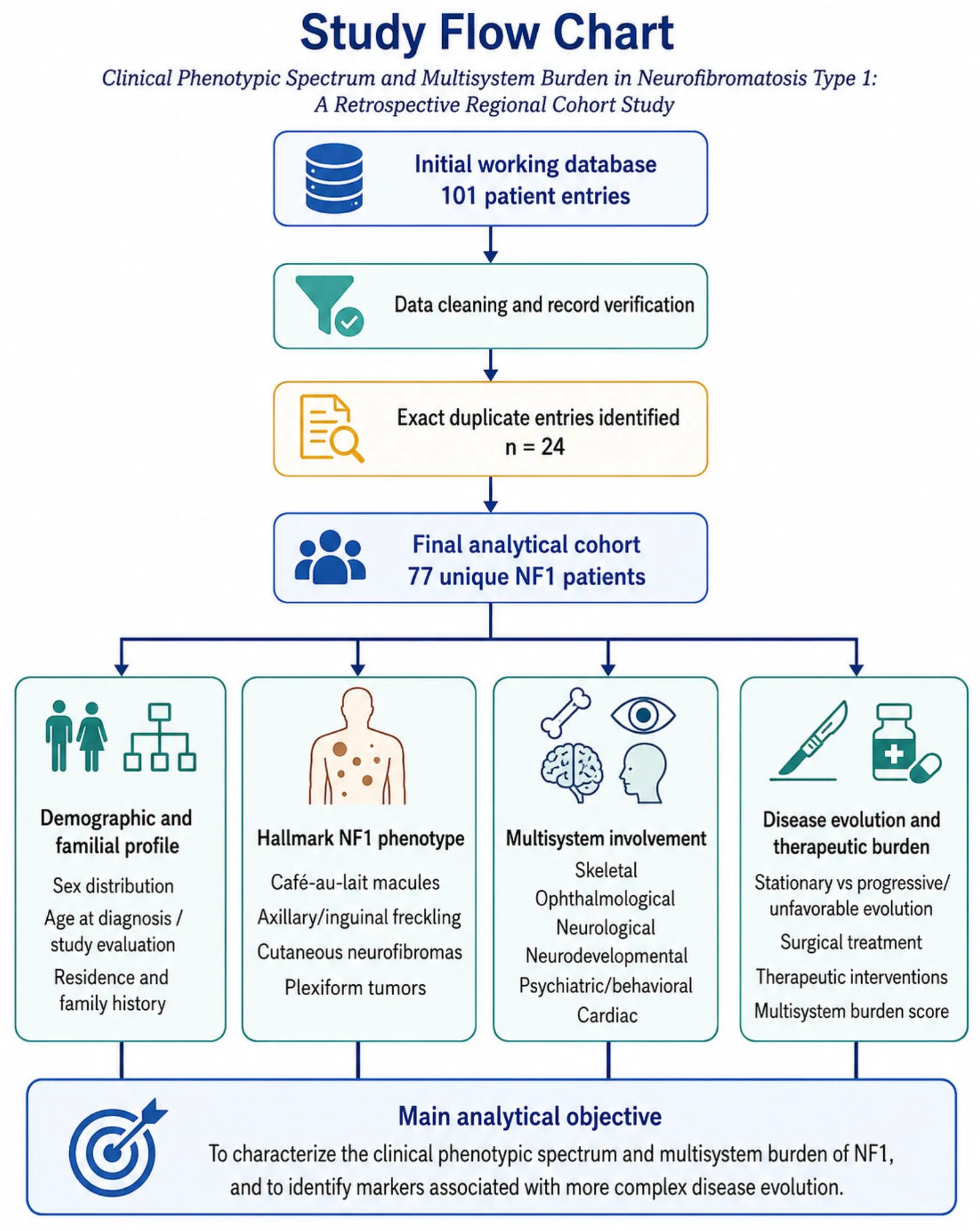
Study flow chart. Café-au-lait macules (CALMs), light brown, well-circumscribed hyperpigmented skin patches, are a hallmark clinical feature of neurofibromatosis type 1 (NF1).

The study flow chart is presented in Figure 1.

## 3. Results

### 3.1. Cohort Characteristics and Familial Background

The final cohort comprised 77 patients, with a slight female predominance (59.7%). The median age at diagnosis was 8.0 years, and 72.7% of patients were diagnosed before adulthood. At study evaluation, the median age was 22.5 years (IQR 14.0–39.3), with a balanced distribution between urban and rural residence. A positive family history was documented in 81.8% of patients, although pedigree linkage between affected relatives was unavailable. Two deaths (2.6%) were recorded during the study period.

Most patients were diagnosed during childhood, with over one-third receiving the diagnosis before 5 years of age. Educational status reflected the mixed pediatric and adult composition of the cohort and is summarized together with the remaining demographic characteristics in Table 1.

**Figure 2:**
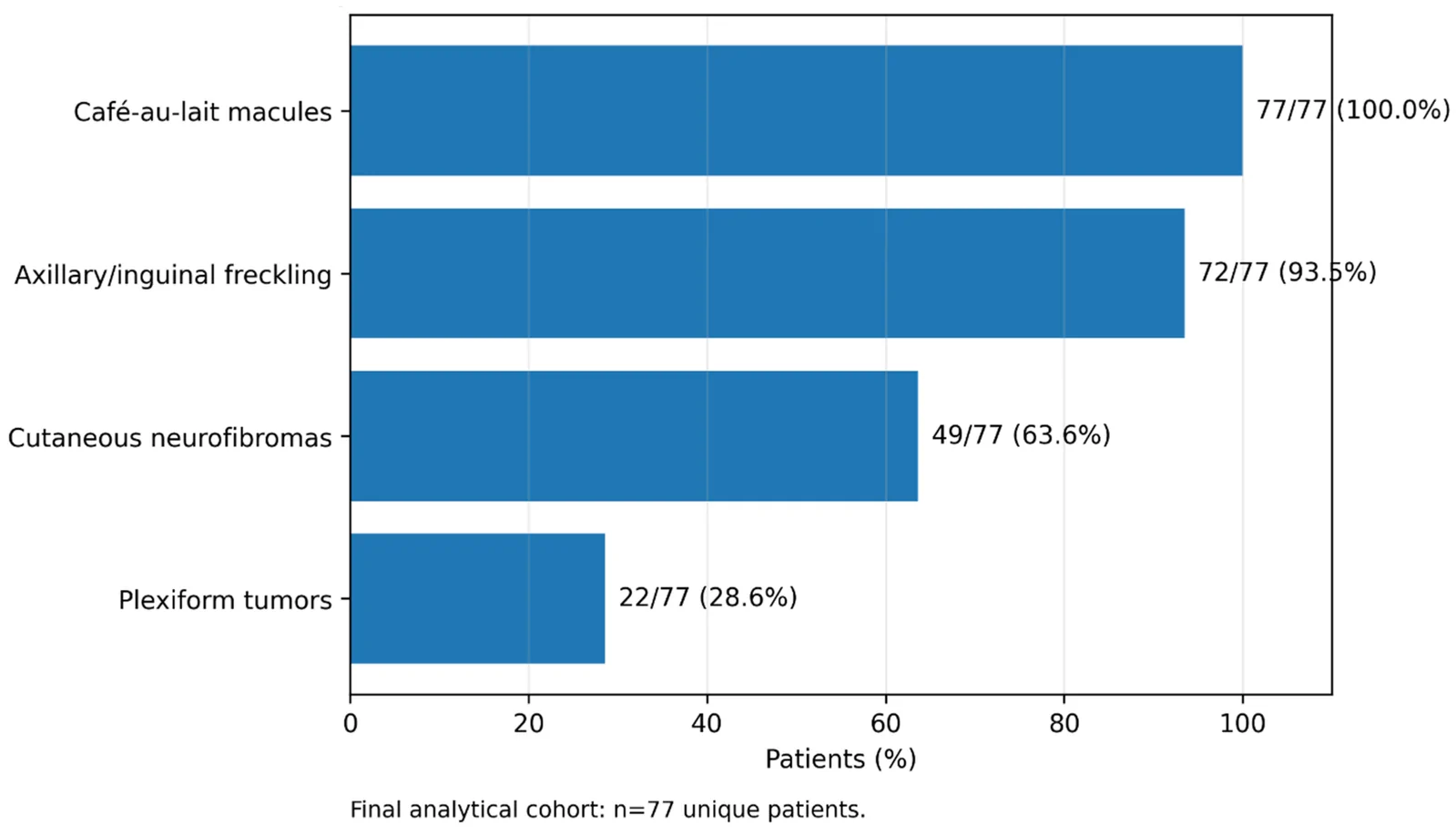
Distribution of hallmark cutaneous and tumor-related manifestations in the NF1 cohort. Data are presented as percentages of the final analytical cohort of 77 unique patients. Plexiform tumors were defined using either direct tumor coding or positive localization/count-related tumor variables. Café-au-lait macules (CALMs), named after the French term meaning “coffee with milk” because of their characteristic light-brown color, are among the hallmark cutaneous manifestations of NF1.

The principal cutaneous and tumor-related findings are summarized in Figure 2 and Table 2.

### 3.2. Cutaneous Hallmark Manifestations and Tumor-Related Phenotype

CALMs were present in all patients, with most exhibiting more than 10 lesions and onset before 10 years of age. Axillary/inguinal freckling was observed in 93.5%, cutaneous neurofibromas in 63.6%, and plexiform tumors in 28.6% of the cohort. Neurofibromas and plexiform tumors were predominantly located on the thorax and extremities; detailed distributions, lesion burden, and age at onset are presented in Table 2.

**Figure 3:**
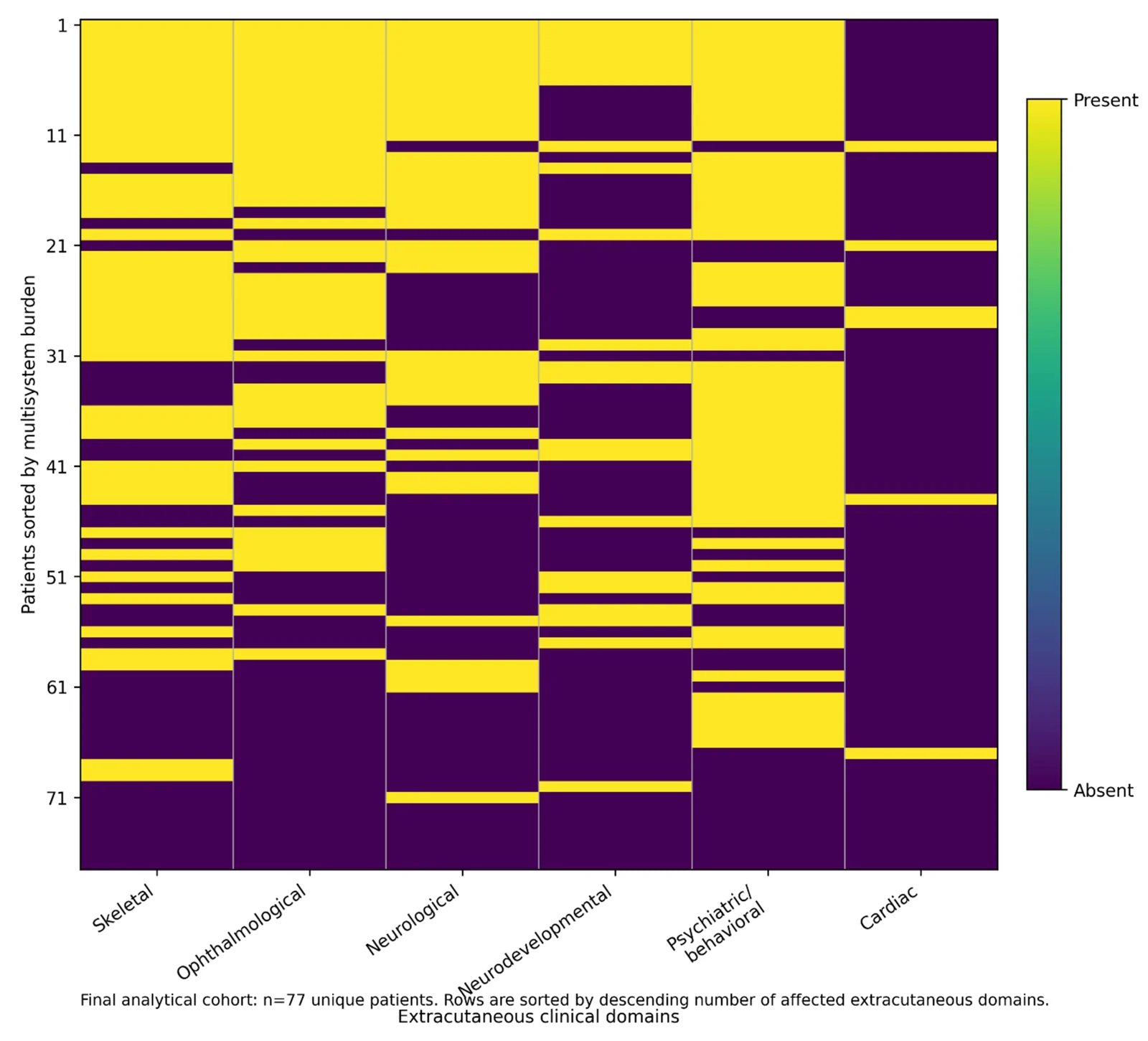
Patient-level heatmap of extracutaneous involvement. Each row represents one individual patient (*n* = 77), ordered from the highest to the lowest multisystem burden score; row numbers are anonymized patient positions rather than clinical categories. Columns represent the six score domains: skeletal, ophthalmological, neurological, neurodevelopmental, psychiatric/behavioral, and cardiac involvement. Yellow indicates documented involvement and purple indicates no documented involvement.

Overall, the cohort exhibited the characteristic pigmentary phenotype of NF1 together with a clinically relevant subgroup affected by plexiform tumors. Figure 3 illustrates the patient-level pattern of extracutaneous domain involvement and is not intended to depict plexiform tumor distribution.

### 3.3. Ophthalmological, Neurological, Skeletal and Multisystem Involvement

Extracutaneous involvement was common in the cohort, particularly psychiatric/behavioral (67.5%), skeletal (57.1%), ophthalmological (51.9%), and neurological manifestations (45.5%), whereas cardiac involvement was uncommon (7.8%). The distribution of specific manifestations and imaging findings is summarized in Table 3.

Lisch nodules were the most common ophthalmological finding, while psychomotor agitation, headache, speech/language disorders, intellectual disability, anxiety, and scoliosis represented the most common neurological, neurodevelopmental, psychiatric, and skeletal manifestations, respectively. Imaging abnormalities were documented in 29.9% of patients, most frequently on MRI and CT examinations (Table 3).

The exploratory multisystem burden score had a mean of 2.57 ± 1.33 affected domains and a median of 3 (range, 0–5). Overall, 92.2% of patients had involvement of at least one extracutaneous domain, and 57.1% had involvement of three or more domains (Table 3; Figure 3).

### 3.4. Therapeutic Burden, Clinical Severity and Disease Evolution

Disease evolution was classified as stationary in 42.9% of patients and as progressive or unfavorable in 57.1%. Overall, 37.7% of patients received at least one therapeutic intervention, most commonly surgery, whereas rehabilitation, symptomatic treatment, and selumetinib were less frequently documented (Table 4). Because lesion-specific treatment indications were inconsistently recorded, therapeutic interventions are presented descriptively and were not interpreted as independent markers of disease progression.

Plexiform tumors were more common in patients with progressive or unfavorable disease compared to those with stationary disease (38.6% vs. 15.2%; *p* = 0.040). They also showed a higher multisystem burden score (median 3 vs. 2), although this difference did not reach statistical significance (*p* = 0.056), and were significantly older both at diagnosis and at study evaluation (Table 5).

Overall, progressive or unfavorable disease was characterized by greater clinical complexity, reflected by a higher frequency of plexiform tumors and a broader pattern of extracutaneous involvement.

Plexiform tumors were more common in the active/adverse group than in the stationary group (38.6% vs. 15.2%, *p* = 0.040). The active/adverse group also had a higher median multisystem burden score (3 vs. 2), although this difference was not statistically significant (*p* = 0.056), and patients in this group were older at evaluation and diagnosis (Table 5). Treatment frequencies are presented descriptively because therapeutic need is intrinsically related to lesion progression and symptoms.

## 4. Discussion

The principal contribution of this study is the application of an exploratory multisystem burden score that summarizes six extracutaneous domains of NF1 into a simple measure of disease breadth. More than half of the patients had involvement of at least three domains, supporting the concept that NF1 extends well beyond its characteristic cutaneous manifestations. Although patients with progressive or unfavorable disease showed higher multisystem scores, this difference did not reach statistical significance and should be considered hypothesis-generating [16,17,18,19,20].

The cutaneous phenotype was consistent with the established clinical spectrum of NF1. CALMs and intertriginous freckling were highly prevalent and support the continued importance of these features for early diagnosis, particularly in childhood, in agreement with current diagnostic recommendations [21,22,23]. The high proportion of patients with a positive family history likely reflects referral patterns and familial clustering, although pedigree linkage was unavailable [24,25].

Plexiform tumors represented one of the most clinically relevant findings. They were significantly more common in patients with progressive or unfavorable disease, supporting previous reports linking these lesions with increased morbidity, functional impairment, and long-term clinical complexity [26,27].

The observed prevalence was likely underestimated because imaging was symptom-driven rather than based on systematic whole-body MRI.

The high frequency of skeletal, ophthalmological, neurological, neurodevelopmental, and psychiatric manifestations further confirms NF1 as a multisystem disorder requiring multidisciplinary management. In particular, the prominent psychiatric and neurodevelopmental burden reinforces recommendations for routine psychological, cognitive, and educational assessment as part of standard NF1 care [28,29,30,31,32,33,34].

Compared with previous cohort studies, the proposed multisystem burden score provides a simple and reproducible method for summarizing cumulative extracutaneous involvement using routinely collected clinical data. However, the score is exploratory, assigns equal weight to all domains, and does not capture disease severity within each organ system. Prospective validation against functional outcomes, quality of life, healthcare utilization, and longitudinal progression is therefore required [35,36].

Treatment data were interpreted descriptively because therapeutic interventions largely reflect disease severity and lesion-specific indications were incompletely documented. The limited use of selumetinib reflects the gradual incorporation of targeted therapies into routine NF1 management rather than treatment efficacy [37,38,39].

Overall, these findings support structured, age-adapted multidisciplinary surveillance, including regular clinical assessment of cutaneous, neurological, skeletal, ophthalmological, and neurodevelopmental manifestations, with imaging guided by clinical indications in accordance with current recommendations [40,41].

The study has several limitations, including its retrospective design, symptom-driven imaging, incomplete lesion-specific treatment information, and the absence of pedigree linkage. In addition, the proposed multisystem burden score has not been externally validated and should currently be regarded as an exploratory research tool.

Despite these limitations, this study provides real-world regional data and introduces a practical framework for describing cumulative extracutaneous involvement in NF1. The findings reinforce the importance of early diagnosis, multidisciplinary follow-up, and individualized risk stratification, while supporting future prospective validation of the proposed multisystem burden score.

## 5. Conclusions

NF1 in this regional cohort showed marked phenotypic heterogeneity and substantial multisystem involvement beyond the characteristic cutaneous manifestations. Plexiform tumors were significantly more common among patients with progressive or unfavorable disease, supporting their role as markers of increased clinical complexity. The proposed exploratory multisystem burden score may facilitate descriptive assessment of disease breadth, although prospective validation is required before clinical implementation.

## Figures and Tables

**Table 1:** Baseline demographic and familial characteristics of the NF1 cohort.

Variable	Result
Final analytical cohort	77 patients
Female sex	46/77 (59.7%)
Male sex	31/77 (40.3%)
Age at diagnosis, years, mean ± SD	15.75 ± 18.17
Age at diagnosis, years, median (IQR)	8.0 (2.0–26.0)
Age at diagnosis, range	0.6–74.0
Diagnosis < 5 years	29/77 (37.7%)
Diagnosis 5–9 years	13/77 (16.9%)
Diagnosis 10–17 years	14/77 (18.2%)
Diagnosis ≥ 18 years	21/77 (27.3%)
Diagnosis before adulthood	56/77 (72.7%)
Valid age at study evaluation	76 patients
Age at study evaluation, years, mean ± SD	27.08 ± 18.13
Age at study evaluation, years, median (IQR)	22.5 (14.0–39.3)
Age at study evaluation, range	1–75
Age at study evaluation < 18 years	31/76 (40.8%)
Age at study evaluation ≥ 18 years	45/76 (59.2%)
Urban residence	40/77 (51.9%)
Rural residence	37/77 (48.1%)
Positive family history	63/77 (81.8%)
No family history documented	14/77 (18.2%)
Deceased patients	2/77 (2.6%)

**Table 2:** Cutaneous hallmark manifestations and tumor-related phenotypes in the NF1 cohort.

Variable	Result
Final ana77.	77 patients
Café-au-lait macules	77/77 (100%)
>10 CALM lesions	70/76 (92.1%)
5–10 CALM lesions	6/76 (7.9%)
CALM onset < 5 years	39/77 (50.6%)
CALM onset 5–10 years	31/77 (40.3%)
CALM onset before 10 years	70/77 (90.9%)
Axillary/inguinal freckling phenotype	72/77 (93.5%)
Axillary freckling	71/77 (92.2%)
Inguinal freckling	64/77 (83.1%)
Neurofibromas present	49/77 (63.6%)
Thoracic neurofibromas	45/49 (91.8%)
Upper-limb neurofibromas	40/49 (81.6%)
Lower-limb neurofibromas	37/49 (75.5%)
Abdominal neurofibromas	31/49 (63.3%)
Neurofibroma onset 10–20 years	31/49 (63.3%)
Neurofibroma burden ≥ 20 lesions	26/49 (53.1%)
Plexiform tumors present	22/77 (28.6%)
Thoracic plexiform tumors	15/22 (68.2%)
Upper-limb plexiform tumors	11/22 (50.0%)
Lower-limb plexiform tumors	6/22 (27.3%)
Single plexiform tumor	5/22 (22.7%)
1–5 plexiform tumors	16/22 (72.7%)
5–10 plexiform tumors	1/22 (4.5%)

**Table 3:** Extracutaneous and multisystem involvement in the NF1 cohort.

	*n*/N (%)
Skeletal involvement, any	44/77 (57.1%)
Scoliosis/other skeletal abnormalities	44/77 (57.1%)
Spina bifida	4/77 (5.2%)
Fractures	1/77 (1.3%)
Genu varum	0/77 (0.0%)
Genu valgum	0/77 (0.0%)
Ophthalmological involvement, any	40/77 (51.9%)
Lisch nodules	25/77 (32.5%)
Optic pathway glioma	4/77 (5.2%)
Glaucoma	2/77 (2.6%)
Other ophthalmological abnormalities	18/77 (23.4%)
Neurological involvement, any	35/77 (45.5%)
Psychomotor agitation	18/77 (23.4%)
Headache	14/77 (18.2%)
Hydrocephalus	2/77 (2.6%)
Epilepsy	2/77 (2.6%)
Intraspinal neurofibromas	2/77 (2.6%)
Radiculopathies	2/77 (2.6%)
Paresis	2/77 (2.6%)
Meningiomas	3/77 (3.9%)
Polyneuropathy	1/77 (1.3%)
Neurodevelopmental involvement, any	21/77 (27.3%)
Speech/language disorders	15/77 (19.5%)
Intellectual disability	13/77 (16.9%)
ADHD	1/77 (1.3%)
Autism spectrum disorder	0/77 (0.0%)
Psychiatric/behavioral involvement, any	52/77 (67.5%)
Anxiety	40/77 (51.9%)
Behavioral disorders	23/77 (29.9%)
Depression	2/77 (2.6%)
Cardiac involvement, any	6/77 (7.8%)
Hypertension	5/77 (6.5%)
Arterial stenosis	1/77 (1.3%)
Other cardiac abnormalities	1/77 (1.3%)
Congenital heart disease	0/77 (0.0%)
Hypertrophic cardiomyopathy	0/77 (0.0%)
Any positive imaging abnormality	23/77 (29.9%)
CT abnormalities	15/77 (19.5%)
MRI abnormalities	17/77 (22.1%)
Abdominal ultrasound abnormalities	8/77 (10.4%)
Multisystem burden score, mean ± SD	2.57 ± 1.33 domains
Multisystem burden score, median, range	3, range 0–5
≥1 extracutaneous domain involved	71/77 (92.2%)
≥3 extracutaneous domains involved	44/77 (57.1%)

**Table 4:** Therapeutic burden and disease evolution in the NF1 cohort.

Variable	Result
Final analytical cohort	77 patients
Stationary evolution	33/77 (42.9%)
Progressive evolution	38/77 (49.4%)
Unfavorable evolution	6/77 (7.8%)
Favorable evolution	0/77 (0.0%)
Active/adverse evolution: progressive or unfavorable	44/77 (57.1%)
Surgical treatment documented	26/77 (33.8%)
Any documented surgical intervention	23/77 (29.9%)
Single surgical intervention	11/77 (14.3%)
1–5 surgical interventions	12/77 (15.6%)
>5 surgical interventions	0/77 (0.0%)
Symptomatic treatment	4/77 (5.2%)
Physiokinetotherapy/rehabilitation	12/77 (15.6%)
Selumetinib treatment	3/77 (3.9%)
Any therapeutic intervention	29/77 (37.7%)

**Table 5:** Clinical characteristics and exploratory multisystem burden according to disease evolution.

Variable	Stationary Evolution *n* = 33	Active/Adverse Evolution *n* = 44	*p*-Value
Plexiform tumors	5/33 (15.2%)	17/44 (38.6%)	0.0401
Multisystem burden score, mean ± SD	2.21 ± 1.43	2.84 ± 1.20	0.056
Multisystem burden score, median	2	3	0.056
High multisystem burden (≥3 domains)	16/33 (48.5%)	28/44 (63.6%)	0.245
Age at study evaluation, years, mean ± SD	19.27 ± 14.86	33.07 ± 18.28	0.0007
Age at study evaluation, years, median	16.0	32.0	0.0007
Age at diagnosis, years, mean ± SD	10.09 ± 13.75	19.99 ± 20.00	0.0244
Age at diagnosis, years, median	4.0	10.5	0.0244

## Data Availability

The data presented in this study are available on request from the corresponding author. The data are not publicly available due to privacy or ethical restrictions.

## Abbreviations

The following abbreviations are used in this manuscript:

Abbreviation	Definition
CALMs	Café-au-lait macules
NF1	Neurofibromatosis type 1
NGS	Next-generation sequencing
MLPA	Multiplex ligation-dependent probe amplification
FISH	Fluorescence in situ hybridization
VUS	Variant of uncertain significance
HGVS	Human Genome Variation Society
MEK	Mitogen-activated protein kinase kinase
SD	Standard deviation
IQR	Interquartile range
